# Label‐Free Nanoimaging of Neuromelanin in the Brain by Soft X‐ray Spectromicroscopy

**DOI:** 10.1002/anie.202000239

**Published:** 2020-05-14

**Authors:** Jake Brooks, James Everett, Frederik Lermyte, Vindy Tjendana Tjhin, Samya Banerjee, Peter B. O'Connor, Christopher M. Morris, Peter J. Sadler, Neil D. Telling, Joanna F. Collingwood

**Affiliations:** ^1^ School of Engineering University of Warwick Coventry UK; ^2^ School of Pharmacy and Bioengineering Keele University Stoke-on-Trent UK; ^3^ Department of Chemistry University of Warwick Coventry UK; ^4^ Newcastle Brain Tissue Resource, Translational and Clinical Research Institute Newcastle University Newcastle-upon-Tyne UK

**Keywords:** iron, neuromelanin, Parkinson's disease, synchrotron imaging, x-ray absorption spectroscopy

## Abstract

A hallmark of Parkinson's disease is the death of neuromelanin‐pigmented neurons, but the role of neuromelanin is unclear. The in situ characterization of neuromelanin remains dependent on detectable pigmentation, rather than direct quantification of neuromelanin. We show that direct, label‐free nanoscale visualization of neuromelanin and associated metal ions in human brain tissue can be achieved using synchrotron scanning transmission x‐ray microscopy (STXM), through a characteristic feature in the neuromelanin x‐ray absorption spectrum at 287.4 eV that is also present in iron‐free and iron‐laden synthetic neuromelanin. This is confirmed in consecutive brain sections by correlating STXM neuromelanin imaging with silver nitrate‐stained neuromelanin. Analysis suggests that the 1s–σ* (C−S) transition in benzothiazine groups accounts for this feature. This method illustrates the wider potential of STXM as a label‐free spectromicroscopy technique applicable to both organic and inorganic materials.

## Graphical abstract

**Soft X‐ray spectromicroscopy** enables neuromelanin visualization in human brain tissue. This removes dependence on optical evaluation of pigmentation or chemical staining, opening the door to investigating relationships between depigmentation, metal binding, and neurodegeneration in the most vulnerable neurons in Parkinson's disease. (Image of brain adapted, under license CCBY4.0, from Blausen.com staff, *WikiJournal of Medicine* **2014**, *1*, ISSN 2002–4436.)

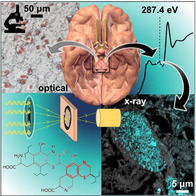

## Introduction

Visualization of neuromelanin (NM), a biological polymer formed by autoxidation of dopamine and cysteine within dopamine and noradrenaline synthesizing neurons, historically relied on the presence of visible pigmentation, with or without staining enhancement. In 1988, Hirsch and co‐workers highlighted how this, by definition, restricts NM visualization to forms of NM with detectable pigment, thereby excluding clusters that are too faint to view using light microscopy.^1^ This need for a neuromelanin‐specific marker has not been met in over three decades, with studies continuing to depend on the natural contrast of the NM pigment viewed under an optical or electron microscope.^2^ This constrains objective investigation of NM in human health and disease, and in other species in which pigmentation is less apparent, carrying an implicit suggestion that the quantity of NM is simply proportional to the detectable pigmentation.

In the human brain, dopaminergic neurons within the substantia nigra normally show pigmentation with advancing age as granules of NM. These neurons are particularly vulnerable during the progression of Parkinson's disease (PD)^1^ and related disorders. In PD, loss of pigmentation is particularly evident in the substantia nigra pars compacta and is directly associated with progressive neuronal loss. PD is the second most common neurodegenerative disease globally, and remains incurable, whilst the underlying causes are still unknown.^3^ By the time a patient's symptoms become apparent, 80 % of dopaminergic neurons may have already died. The dopamine depletion resulting from death of the substantia nigra neurons gives rise to the typical clinical symptoms observed for PD patients, including tremor, rigidity, and bradykinesia. Whilst no consensus has been reached regarding any biological function of NM, and a direct relationship between neuron vulnerability and the presence of NM remains unclear, the strong affinity of NM for metal ions is a factor receiving considerable attention.^4^

NM has been proposed as the main chelator of neuronal iron in the substantia nigra, and 50‐fold increased concentrations of iron compared to surrounding tissue have been observed.^5^ NM has been shown to sequester Fe^2+^ ions and bind them as Fe^3+^, creating iron oxide clusters, which comprise approximately 10–20 % of total iron in the substantia nigra.^6^ The remaining iron is mostly stored by ferritin in the glia.^6^ Since PD is associated with a marked increase in nigral iron concentration,^7^ it is imperative to discern the individual roles played by these two iron storage compounds if normal physiological iron concentration is exceeded.

**Figure 1:**
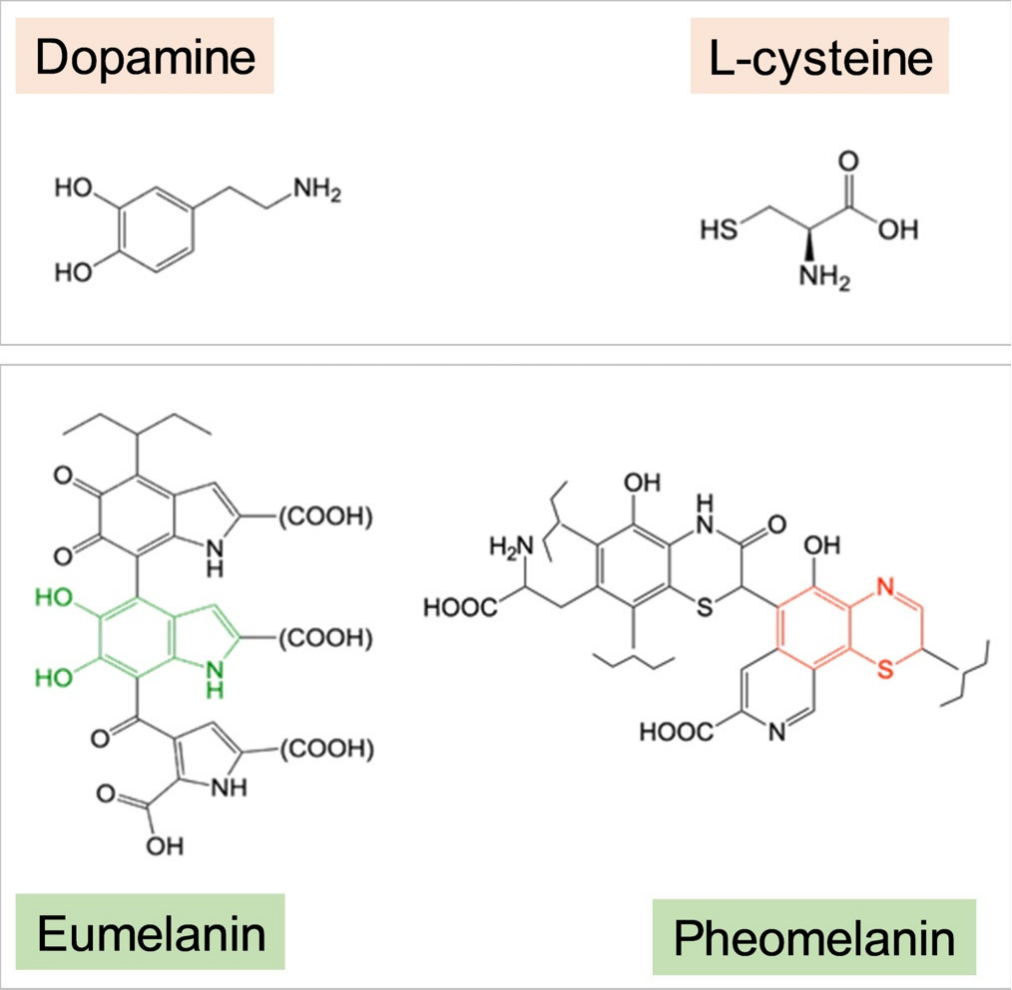
Structures of the neuromelanin precursors, dopamine and l‐cysteine, alongside partial structures of eumelanin (NM surface) and pheomelanin (NM core). Dihydroxyindole=green and benzothiazine=red.

Within the brain, NM is contained within organelles 0.5–3 μm in size, along with proteins, lipid bodies, and bound metal ions. NM clusters in the substantia nigra range from 200–600 nm in size, and are divided into spherical granules of circa 30 nm diameter.^8^ Diffraction studies indicate that the NM polymer itself consists of a central protein core with a similar structural motif to β‐pleated sheets of amyloid aggregates (β strands that run perpendicular to the fiber axis, forming a cross‐β sheet), surrounded by an unstructured melanic polymer.^6^, 7c, 9 The melanic portion can itself be divided into two structural components. Pheomelanin is formed first by oxidative co‐polymerization of cysteine and dopamine and characterized by benzothiazine groups. Once cysteine levels have been depleted, slower oxidative homopolymerization of dopamine results in the formation of eumelanin, which is characterized by dihydroxyindole groups and forms at the surface of the pheomelanin.^10^ Partial structures for eumelanin and pheomelanin are displayed in Figure 1, alongside structures of the precursors dopamine and l‐cysteine.

The ability of NM to sequester iron and other metals in a non‐reactive form is thought to act as an intrinsic defense against labile metal ion accumulation. Binding of metal ions to the insoluble NM restricts their participation in extracellular redox activity, thereby shielding surrounding cells from oxidative stress and associated toxicity.7d, 9 This putative protective role of NM is consistent with its accumulation in the brain during ageing, its affinity for toxic compounds, and its ability to remove cytosolic catechols.4b Conversely, iron released by NM into the extra‐cellular milieu may stimulate oxidative stress mechanisms, contributing to the selective neurodegeneration of pigmented neurons. If NM is released from the cell upon cell death, NM–iron complexes may activate microglia to release cytokines and neurotoxins, inducing a vicious cycle of chronic inflammation and neuronal loss in the PD substantia nigra.7d, 9, 11 Thus it has been hypothesized that NM may play a protective or destructive role depending on cellular context.^9^

Synthetic models of NM have been explored by several groups.^12^ Such analogues are essential, since isolating NM from substantia nigra tissue is highly challenging and yields only small quantities.12b Wider investigation into the dual protective/damaging role of NM is reliant on in vitro and ex vivo studies being carried out with sufficient sensitivity to detect chemical or structural changes relating to NM–metal binding. This of course depends a priori on the ability to identify accurately NM in brain tissue. Histochemical techniques are traditionally used for melanin visualization, including the DOPA oxidase, ferrous iron, and Masson‐Fontana methods.^13^ The propensity of NM to be labelled using silver stains, similar to other melanins, is indeed partly responsible for its original melanin classification.^14^ Numerous studies have identified the presence of NM using the Masson‐Fontana silver stain,2b, 15 which causes melanin to appear black as it reduces ammoniacal silver nitrate to metallic silver.^16^ However, for trace metal analysis, staining is an unsuitable mapping technique, as it alters the native chemistry and prohibits the accurate speciation of associated metal ions. Contrast agents routinely used to prepare samples for microscopic imaging also eliminate the potential for in situ chemical analysis.

Good sample preservation is essential for optimal chemical imaging analysis of delicate biological materials. A key advantage of x‐ray imaging is that significantly reduced beam damage is expected from x‐ray beam exposure compared to electron beam exposure at equivalent spatial resolution.^17^ Additionally, whilst conventional electron microscopy may be hampered by over‐sensitivity to electron‐dense material, the greater penetrating power of x‐ray microscopy is beneficial for characterizing the fine structure of electron‐dense materials such as melanin granules.^18^

Scanning transmission x‐ray microscopy (STXM) is a synchrotron‐based spectromicroscopy technique that combines excellent chemical sensitivity with high‐resolution microscopy. Usually employed in the soft x‐ray range (circa 0.1–1 keV) for biological samples, this approach permits access to both organic and inorganic (metal) absorption edges without the necessity for artificial staining (typically required for light or electron microscopy).^17^, ^19^ Soft x‐ray microscopy is capable of spanning the water window (280–540 eV range), in which there is an order of magnitude difference between absorption coefficients for water and protein.

In STXM, monochromatic x‐rays are focused into a nanometer‐sized spot (pixel) on the sample using a zone plate. A microscopic image is generated by raster scanning the sample in the focal plane of the zone plate whilst measuring transmitted x‐ray intensity for each pixel. When the incident x‐ray energy is stepped over a range (typically over a targeted absorption edge), spectra can be obtained, providing information about the sample composition. STXM is a technique that has benefited from vast improvements in beam focusing and detector sensitivity, such that it is now capable of characterizing chemical composition over 50 nm‐sized areas at sub‐part‐per‐million sensitivity.^17^ Thin sections used for STXM (100–200 nm) permit spectra to be collected at the carbon *K*‐edge, allowing metal speciation to be correlated with protein distribution. This has been previously demonstrated to good effect in detailed characterizations of amyloid‐β/iron aggregates formed in vitro,^20^ ex vivo human brain tissue,^19^ and amyloid plaque cores extracted from Alzheimer's disease brain tissue.^21^

In addition, the ability to generate image contrast artificially by exploiting specific spectral features, uniquely allows the technique to be used to map selectively structures for which histological stains do not exist. This was exploited recently by using spectral features measured at the carbon *K*‐edge, to map variations observed when extracellular peptides were bound to iron in cortical tissue from a transgenic mouse model of Alzheimer's disease.^22^

Herein, we establish the potential of STXM for label‐free mapping of NM in human brain substantia nigra pars compacta tissue from both PD and non‐PD cases, and evaluate NM–metal binding. Method development is informed with x‐ray absorption spectra from reference materials to assess signal specificity and likely origin of spectral features associated with NM.

To date, advances in understanding PD etiology have been hampered by a lack of tools for investigating interactions between organic and inorganic (metal ion) phases in the PD brain, since most methods demand a mutually exclusive choice be made between performing an analysis of the organic or inorganic properties of a particular tissue sample. In recognizing the importance of this system, Biesemeier and co‐workers noted in 2016 the surprising lack of high‐resolution chemical analysis and imaging studies to investigate the interaction between NM and iron.^8^ Herein, we show that soft x‐ray spectromicroscopy can address this technical challenge, and provide new insights into the metallomics of PD and related disorders.

## Results

Speciation maps and x‐ray absorption spectra were collected from resin‐embedded substantia nigra tissues at the carbon *K*‐edge, focusing on NM‐rich regions within dopaminergic neurons and regions of tissue adjacent to the neurons (neuropil). Full procedures for generating speciation maps and absorption spectra are shown in Supporting Information Figures S1 and S2, respectively. A particular advantage of STXM, as illustrated in Figure S1, is the capacity to display signal(s) from analytes of interest (e.g., neuromelanin, Figure S1 c, and protein, Figure S1 e) without interference from artefacts such as score marks from tissue sectioning. Carbon *K*‐edge absorption spectra were also collected from resin‐embedded in vitro standards, alpha‐synuclein, ferritin, synthetic NM (with and without iron‐loading), and benzothiazine, in order to evaluate contributions to the tissue spectra.

**Figure 2:**
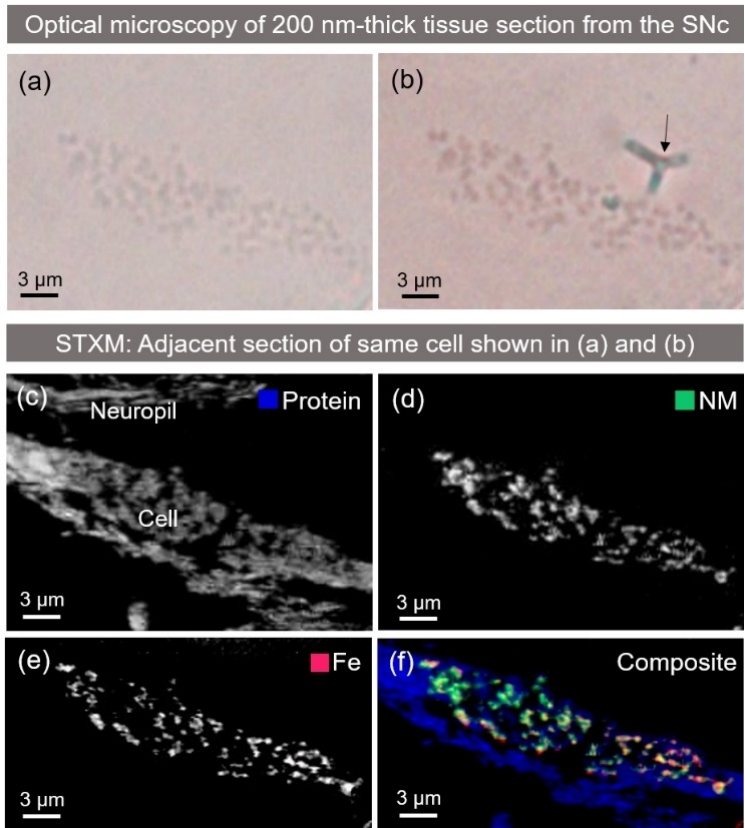
Dopaminergic neuron identified in PD (case PD1) substantia nigra pars compacta (SNc) tissue section (200 nm thickness) using optical microscopy, then mapped using STXM at the carbon *K*‐edge and Iron *L*‐edge in a consecutive tissue section. a) Unstained cell, b) Masson‐Fontana stained cell, arrow marks the position of a fold introduced into the resin by staining, c) protein speciation map, d) NM speciation map using peak at 287.4 eV, e) iron map, f) composite map showing protein (blue), NM (green) and iron (red). The individual RBG color channels were manually adjusted for display purposes.

Optical images were initially used to locate dopaminergic neurons within substantia nigra tissue prior to STXM analysis, see Figure 2 a,b. The NM pigment was just visible without staining, with enhanced contrast appearing after staining.

For case PD1 (Supporting Information Table S1), the same cell shown in Figure 2 was successfully identified by optical microscopy in a consecutive, unstained section and mounted for STXM analysis. Scanning at 287 eV, a NM‐rich region within the cell was selected, guided by the optical micrograph of the paired stained section shown in Figure 2 b. A neuropil region was selected by first creating a protein speciation map; an off‐peak image (290.5 eV) was subtracted from a peak image taken at 288.6 eV, corresponding to an amide 1s→π* transition in tissue‐derived protein. The resultant map displayed the tissue ultrastructure within the section, as shown in Figure 2 c.

**Figure 3:**
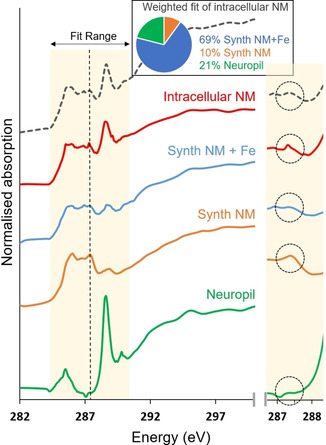
Carbon *K*‐edge *x*‐ray absorption spectra from intracellular NM in PD substantia nigra (case PD1), synthetic NM, synthetic NM+Fe and neuropil region, highlighting an absorption feature that enables specific isolation of NM signal from surrounding tissue without staining. Traces are vertically offset for clarity. A dotted line marks the position of the absorption feature attributed to NM at 287.4 eV, which is not observed within the NM‐free tissue region. Results from linear combination fitting of the intracellular NM spectrum are over‐plotted with a dashed line. Intracellular NM was consistent with 21 % protein and 79 % NM (69 % NM+Fe, 10 % NM), with χ^2^ value of 0.04 obtained for the fit. The difference between NM and NM+Fe is sufficient to affect fit quality, but we note (as evidenced in the individual traces shown), the differences between NM and NM+Fe arise predominantly from the relative amplitude of features rather than presence or absence of peaks in the energy spectrum. An expanded view of the feature in the energy region 286.5–288.5 eV is shown in the panel on the right.

A series of images (“stack”) across the carbon *K*‐edge (280–320 eV) was acquired within both NM‐rich and neuropil regions in PD substantia nigra (Figure 3) in order to compare the absorption spectra. The spectrum acquired from the neuropil region was defined by three clear peaks at 285.5, 288.6, and 290 eV. The peaks at 285.5 and 288.6 eV can be attributed to the 1s→π* transitions for aromatic and amide groups, respectively.^23^ The peak at 289.8 eV can be attributed to 1s→σ*_(C‐N)_ transitions in various amino acids, including arginine and asparagine.^24^ The spectrum acquired from the intracellular NM region exhibited suppression of the principal amide peak (288.6 eV) compared to protein, and the appearance of a doublet feature, including a characteristic peak at 287.4 eV that was not observed in the tissue spectrum, but that was also found in the synthetic NM spectra. Spatial resolution of 100 nm enabled individual NM granules to be easily discerned from surrounding tissue at 287.4 eV (Figure S2).

An additional speciation map was created by subtracting an off‐peak image (290.5 eV) from an image acquired at 287.4 eV, where the characteristic NM absorption feature was observed. This is shown in Figure 2 d. By acquiring large‐area STXM maps of the whole cell body at 200 nm spatial resolution, a detailed depiction of granular morphology and distribution of NM could be resolved.

Correlation analysis of an optical image acquired from a Masson‐Fontana stained section (Figure 2 b), and a STXM NM speciation map in the corresponding adjacent section (Figure 2 d) confirmed a spatial correlation between the optical and synchrotron‐measured cellular distributions of NM. The correlation coefficient was 0.71 for the cell body, excluding the region with the folding artefact (Supporting Information Figure S3).

The same neuron from case PD1 was further analysed at the Fe *L_3_*‐absorption edge to examine the distribution of iron within NM granules. The cell was shown to be heavily loaded with iron (Figure 2 e), with a distribution closely matching that of NM. Cross‐correlation of the entire cell body area in the synchrotron‐acquired NM and iron maps yielded a correlation coefficient of 0.76 (Figure S3), and for the spatial region defined in Figure S3 a,b the correlation coefficient for synchrotron‐acquired NM and iron maps was equivalent (0.75). Additional examples of correlative staining and NM mapping are shown for a neurologically healthy control case in Figures S4 and S5.

Carbon *K*‐edge absorption spectra collected from both iron‐free and iron‐loaded synthetic NM analogues are shown alongside intracellular NM from PD1 in Figure 3. The absorption feature at 287.4 eV was reproduced in both the intracellular and synthetic NM spectra. Linear combination fitting of the intracellular NM spectrum from case PD1 was performed to ascertain how well the spectrum could be explained in terms of contributions from tissue‐derived protein and synthetic NM (i.e., pure dopamine/cysteine polymer). Fitting was limited to the energy range between 283.5 and 290.5 eV in order to focus on the features of interest. A weighted fit of the intracellular NM spectrum was consistent with 21 % tissue‐derived protein and 79 % NM (69 % iron‐loaded synthetic NM, 10 % iron‐free synthetic NM).

**Figure 4:**
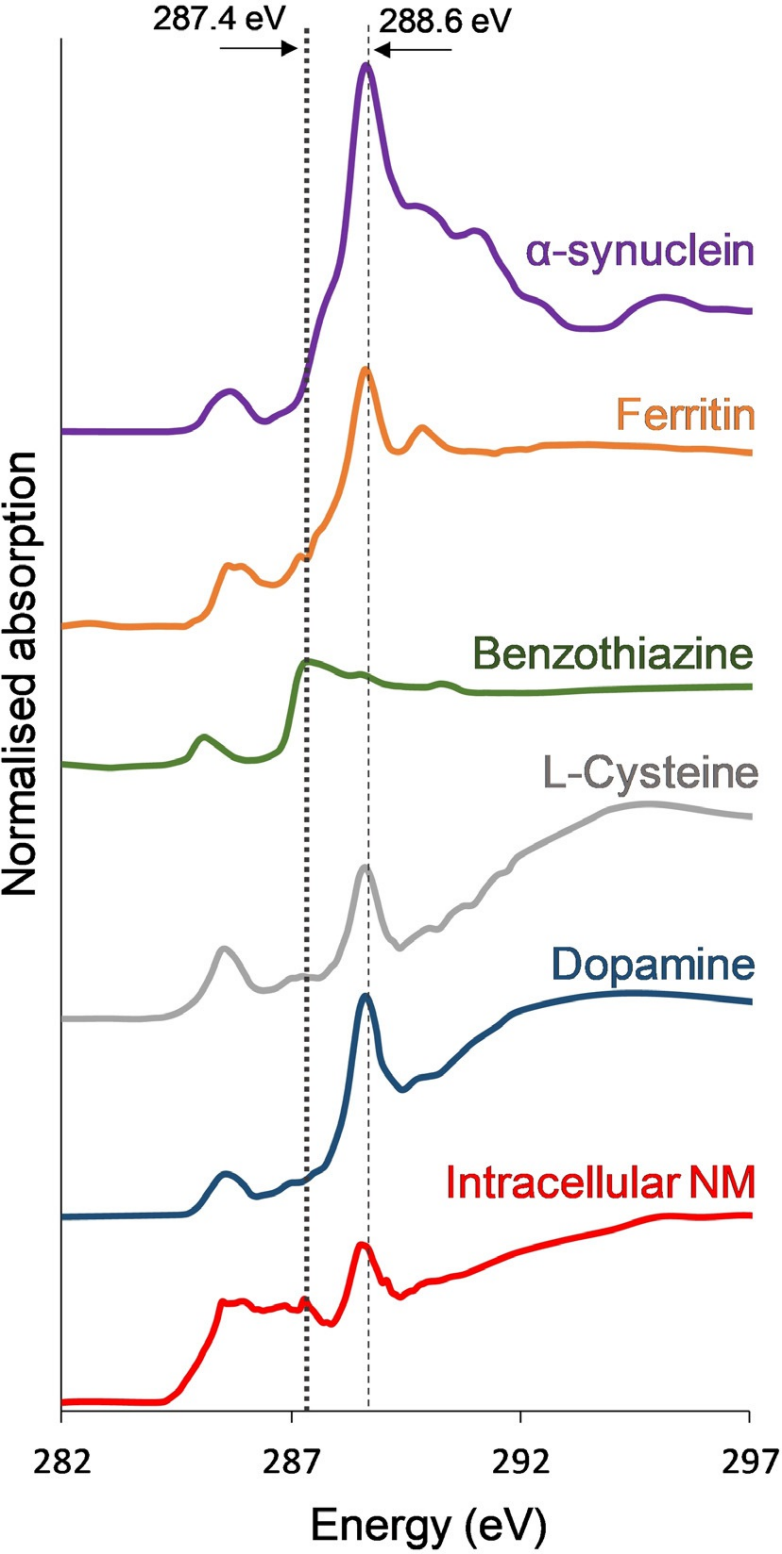
Carbon *K*‐edge *x*‐ray absorption spectra from intracellular NM, precursors dopamine and l‐cysteine, intra‐cellular components ferritin and alpha‐synuclein, and NM functional group benzothiazine. A dotted line marks the position of the absorption feature distinguishing NM at 287.4 eV, shown to be dominant in the benzothiazine spectrum, but absent in the other presented carbon spectra. A dashed line marks the position of the principal amide peak at 288.6 eV. Spectra acquired for all samples shown (except intracellular NM and ferritin) were subjected to 3‐point smoothing for display purposes to accommodate lower signal‐to‐noise above 290 eV. Traces vertically offset for clarity.

Figure 4 shows carbon *K*‐edge absorption spectra acquired from precursors dopamine and l‐cysteine alongside the spectrum for intracellular NM, previously shown in Figure 3.

Spectra for benzothiazine, which occurs as a functional group within the pheomelanin structure (see Figure 1), as well as the intracellular proteins alpha‐synuclein and ferritin are also compared. It can be seen that the overall NM spectrum is distinct from those of its precursor materials. However, a broad feature centered at 287.4 eV is evident in the spectrum of l‐cysteine and a much sharper absorption feature is present in the benzothiazine spectrum at 287.4 eV, precisely matching the energy position of the characteristic NM absorption feature.

**Figure 5:**
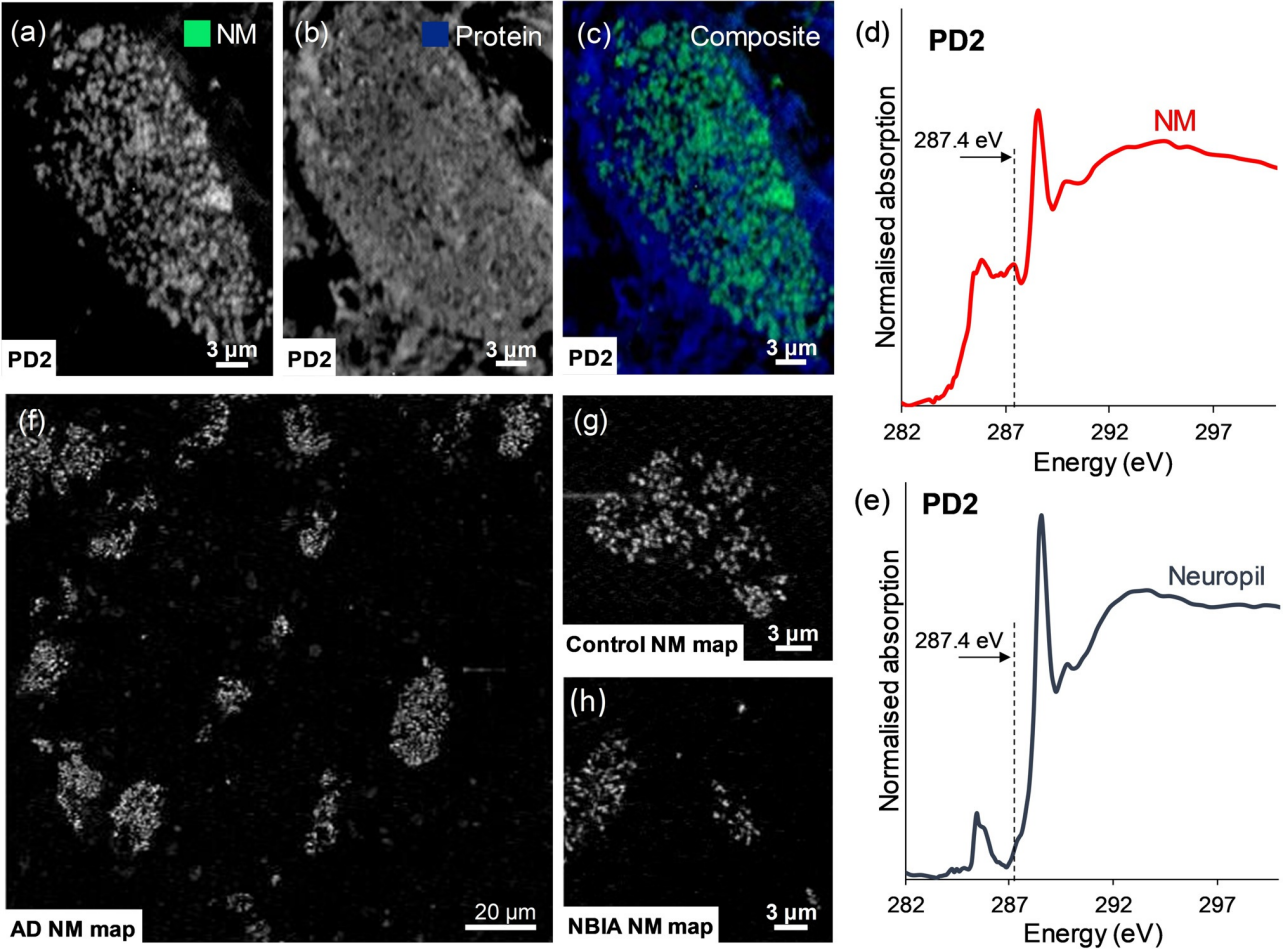
STXM analysis of case PD2, in which for a single neuron, speciation maps show distributions of a) NM, b) protein, c) composite map (NM=green, protein=blue). Carbon K‐edge spectra were also acquired from d) intracellular NM and e) neuropil regions to verify reproducibility of the distinct spectral features observed in PD1 (Figure 3). (f)–(h) show application of NM mapping to post‐mortem human substantia nigra tissue as described in Table S1: f) Alzheimer's disease (AD) case, showing NM distribution over large field of view including multiple pigmented neurons; g) neurologically healthy case (Control), showing NM contrast associated with a single neuron; h) neurodegeneration with brain iron accumulation (NBIA) case, showing contrast associated with neuromelanin deposits in the tissue.

Having discovered and described the spectral feature that permitted NM mapping in PD (case PD1), this spectral feature was confirmed in a second case of PD (PD2), and further applied to NM imaging of post‐mortem tissue from subjects with Alzheimer's disease, neurodegeneration with brain iron accumulation, and a healthy control. The NM contrast was consistently observed in all examples as illustrated in Figure 5.

## Discussion

Comparison of x‐ray absorption spectra obtained from NM‐rich and neuropil regions within substantia nigra tissue revealed differences between NM polymer and tissue‐derived protein. We show how a distinguishing feature present in the NM spectrum at 287.4 eV, but absent in the tissue‐derived protein spectrum, can be exploited for selective label‐free mapping of NM in tissue, without the necessity for chemical staining. Indeed, neuromelanin contrast arising from the spectral feature centered at 287.4 eV was consistently observed in substantia nigra tissue from the five independent cases, irrespective of disease classification (Table S1). This suggested approach was supported by comparing neuromelanin maps obtained with conventional silver nitrate staining and label‐free STXM analysis, in consecutive sections from case PD1 (Figure S3). The high spatial resolution of STXM enabled individual neuromelanin granules to be clearly resolved, such that correlated NM distributions were evidenced both with and without staining.

The structural relevance of the synthetic NM analogues (both with and without iron loading) was demonstrated by comparing their absorption spectra with that obtained from intracellular NM. The same characteristic shape was observed in all three cases, with peaks present at the same energy values. In future, for in vitro studies, STXM could be employed to trace NM distribution in complex model systems for example, in competitive metal binding experiments. Linear combination fitting suggested that intracellular NM is composed of part protein and part NM polymer, consistent with the multiple structural components of intracellular NM reported in the literature.^6^, ^25^

Previously, Biesemeier and co‐workers used NanoSIMS to demonstrate that elevated sulfur levels in NM distinguish NM from surrounding tissue.^8^ In the present work, we mapped x‐ray fluorescence at 200 nm resolution in NM‐rich regions of PD substantia nigra tissue, obtaining maps at the iron and sulfur *K*‐edges as shown in Figure S6. Although sulfur is unambiguously elevated in NM, consistent with the prior observation, sulfur is ubiquitously distributed throughout human brain tissue in amino acid residues cysteine and methionine, as well as other thiols, disulfides, and sulfates. Our STXM work utilizing a signature in the NM spectrum, present in both intracellular and synthetic NM, provides an independent marker. Advantages conferred by this include capacity to differentiate unambiguously deposits of extracellular NM from other material such as concentrated sulfate, and the benefit of simultaneously mapping the surrounding tissue using another absorption feature present in the carbon K‐edge spectrum, allowing both signals to be extracted from a single measurement.

The ability to distinguish NM from surrounding tissue without staining or prerequisite identification of intact cells is highly useful for mapping NM over relatively large (millimeter‐sized) areas; however, STXM also has capacity to focus over micron‐sized regions of interest at nanoscale resolution. The scope for characterization of both organic and inorganic tissue components using STXM over these length scales has been previously demonstrated.^19^, ^21^ Hence, it is important to consider also the potential for distinguishing NM from other intracellular constituents, particularly those with affinity for metal ions. Figure 4 indicates that x‐ray absorption spectroscopy (XAS) at the carbon *K*‐edge may indeed be used to differentiate NM from the iron storage protein, ferritin, and the dominant constituent of Lewy bodies, alpha‐synuclein. Given the relative ease with which soft x‐ray techniques can switch between organic and inorganic (metal) edges, STXM could be further applied to examine NM‐bound metals independent of ferritin‐/synuclein‐bound metals within the same cell. Moreover, the nanoscale resolution of STXM permits characterization of sub‐micron‐sized deposits that may be present at concentrations below the detection limits of alternative techniques,^26^ thus providing a critical opportunity to investigate the individual role played by NM in metal ion metabolism. This area has been largely unexplored by high‐resolution spectromicroscopy,^8^ particularly in unfixed tissue in which chemical alteration is minimized.

The precise origin of each spectral difference between NM and NM‐free tissue cannot be conclusively determined using STXM alone; however, it is possible to speculate based on the standards measured in this study, knowledge of the functional groups present, and XAS data available from the literature. The “building block principle” states that spectra acquired from complex structures can be divided into individual contributions from specific functional groups/bonds.^27^ Important caveats to this approach are the effects of delocalization of electronic charge across functional groups giving rise to new molecular orbitals,^27^ the effects of changing bond length on overlap between orbitals,^28^ and practical restrictions on available energy resolution. Although the number of studies that have collected XAS data from melanin at the carbon *K*‐edge are very limited, comparisons to the present study should be drawn, where possible, to limit the influence of the aforementioned factors.

The peak at 287.4 eV, used to create NM speciation maps, likely originates from the pheomelanin portion of NM, given that previous XAS measurements of eumelanin did not report the presence of features between peaks observed at 285.5 eV (1s→π* aromatics) and 288.6 eV (1s→π* carboxyl groups).^29^ A pronounced peak at 287.4 eV observed in the XAS spectrum of l‐cysteine has previously been ascribed to C−SH bonds, specifically the 1s→σ*(C−S) transition, with the position of the peak being independent of C−S bond length.^27^ The present study confirmed the appearance of a broad feature centered at 287.4 eV in the spectrum of l‐cysteine, and whilst it is probable that this feature contributes to the NM spectrum, Figure 4 demonstrates that neither precursor material (dopamine or cysteine) can fully account for the sharp feature observed at 287.4 eV in the NM spectrum.

Earlier XAS studies of synthetic and intracellular neuromelanin at the sulfur *K*‐edge found that sulfur may be present in a cysteine‐like environment, or contained within a benzothiazine‐like ring (heterocycle containing one sulfur and one nitrogen atom).^30^ Contributions from these functional groups would arise from the core pheomelanin component of neuromelanin (as shown in Figure 1) but would be absent in either dopamine or cysteine independently. We therefore postulate a likely influence of benzothiazine groups on the observed NM spectrum and the characteristic feature at 287.4 eV, used to distinguish NM polymer from surrounding tissue. On testing this, we found that a prominent absorption peak was indeed present in the benzothiazine spectrum at 287.4 eV, thus supporting our hypothesis. We suggest that the 1s→σ* transition (C−S) is the most plausible explanation for this peak in the benzothiazine spectrum, since this transition has been reported to occur around this energy in a range of organic, sulfur‐containing molecules.^24^ Speciation mapping at the carbon *K*‐edge using a peak attributable to the 1s→σ* (C−S) transition in benzothiazine could be further applied to identify peripheral pheomelanin in a range of biological samples, including skin and hair samples.

## Conclusion

We have shown that a characteristic peak in the carbon *K*‐edge x‐ray absorption spectrum of neuromelanin can be used as a basis for label‐free mapping of neuromelanin in human brain tissue. Based on standards and peak assignments reported in the literature, we show that this peak can be assigned to a 1s→σ* (C−S) transition in benzothiazine fragments found in the pheomelanin portion of neuromelanin. Hence, this approach could be applied to selective visualization of pheomelanin in a range of biological samples. This new method holds the following key advantages over conventional staining or electron microscopy techniques: 1) Neuromelanin is visualized without reliance on detectable pigmentation, 2) no chemicals that demonstrably displace or alter the target species are introduced, 3) neuromelanin distribution is isolated from those of other tissue components, 4) artefacts in the tissue (e.g., score marks) are removed upon image subtraction, 5) effective spatial resolution is unaffected by highly electron‐dense deposits, and 6) photo‐induced damage is minimized. Straightforward switching between characteristic edges for light and heavy elements also presents further opportunities to correlate the distributions of neuromelanin with different metals implicated in Parkinson's disease and related disorders, including iron, which was highlighted in this study. This may help shed light on the enigmatic role of neuromelanin.

Data Availability: The data that support the findings in this study are available in the Warwick Research Archive Portal (WRAP) repository, http://wrap.warwick.ac.uk/135050/.

## Conflict of interest

The authors declare no conflict of interest.

## Supporting information

As a service to our authors and readers, this journal provides supporting information supplied by the authors. Such materials are peer reviewed and may be re‐organized for online delivery, but are not copy‐edited or typeset. Technical support issues arising from supporting information (other than missing files) should be addressed to the authors.

Supplementary
