# Supplementary material for: Label‐Free Nanoimaging of Neuromelanin in the Brain by Soft X‐ray Spectromicroscopy
Source: Angew Chem Int Ed Engl. 2020 May 14;59(29):11984–91. doi: 10.1002/anie.202000239 (PMC7383895; doi:10.1002/anie.202000239)
Supplement: Supplementary file 1 — Supplementary [file ANIE-59-11984-s001.pdf]

Angewandte Chemie
Eine Zeitschrift der Gesellschaft Deutscher Chemiker

Supporting Information

Label-Free Nanoimaging of Neuromelanin in the Brain by Soft X-ray Spectromicroscopy

*Jake Brooks, James Everett[+], Frederik Lermyte[+], Vindy Tjendana Tjhin, Samya Banerjee, Peter B. O'Connor, Christopher M. Morris, Peter J. Sadler, Neil D. Telling, and Joanna F. Collingwood**

anie_202000239_sm_miscellaneous_information.pdf

SUPPORTING INFORMATION

Contents

Experimental Methods 1
- **A. Tissue preparation** 1
- **B. Synthesis of neuromelanin analogue** 2
- **C. Reference materials** 2
- **D. Masson-Fontana Staining for melanin** 2
- **E. Optical Imaging** 3
- **F. X-ray spectromicroscopy measurements** 3
- **G. Linear combination fitting and statistical analysis** 4
- **H. X-ray fluorescence mapping** 4
  - **Table S1:** 4
  - **Figure S1:** 5
  - **Figure S2:** 6
  - **Figure S3:** 7
  - **Figure S4:** 8
  - **Figure S5:** 9
  - **Figure S6:** 9
  - **Figure S7:** 10

References 10

Experimental Methods

A. Tissue preparation

Samples of frozen substantia nigra, that had not been chemically fixed, were obtained from the Newcastle Brain Tissue Resource (NBTR) and the Canadian Brain Tissue Bank (CBTB), and prepared and analyzed under current ethical approvals 07/MRE08/12 and REGO-2018-2223. A summary of the cases (PD and non-PD controls), and how they were used, is shown in Supporting Information Table S1.

Tissue samples were cut into cubes (approx. 8 mm^3) using a diamond blade (to prevent metal contamination) and dehydrated through an ethanol series (40% - 100% dry). A six-stage resin-embedding procedure was employed to prepare samples for synchrotron measurements. Trimethylolpropane triglycidyl ether (TTE) and 4 4'-methylenebis 2-methylcyclohexylamine (MMHA) were mixed in a 1:1 molar ratio to produce a custom aliphatic resin free of carbonyl or aromatic groups, thereby avoiding spectral overlap with tissue-derived protein peaks. Tissue samples were initially immersed in 75% dry ethanol, 25% resin. The resin proportion was increased in increments of 25% every two hours with mixing, with two further hourly changes of 100% resin, and then curing overnight in 1.5 ml molds at 60 °C.

WILEY-VCH

Thin sections of resin-embedded substantia nigra were cut to 100 and 200 nm at room temperature with a diamond blade using a Reichert-Jung ultra-cut microtome. Additionally, for case PD1 (Table S1), consecutive sections were taken from tissue blocks to enable correlative staining and STXM analysis of the same cells. Sections intended for staining were dried onto glass microscope slides and stained for neuromelanin using Masson-Fontana silver nitrate stain, as described in Methodology section part D. Sections to be analyzed by STXM were air-dried onto copper TEM grids. Correlation of regions of interest between slide-mounted and grid-mounted sections was carried out using optical microscopy prior to synchrotron analysis. The distinctive shape of the neuromelanin distribution and the score marks produced by the blade enabled the same cells to be identified in stained and unstained sections. The grid position of the cell was noted so that it could be relocated using STXM imaging.

Thicker sections (2 - 3 μm) of resin-embedded substantia nigra were prepared from a single PD case for hard x-ray fluorescence analysis. Sections were air dried onto gold TEM grids.

B. Synthesis of neuromelanin analogue

In order to prepare the synthetic NM analogue, dopamine and L-cysteine in a 6:1 molar ratio were dissolved in 500 ml of 40 mM phosphate buffer (pH 7.4), as described by Shima *et al* [1]. Two parallel batches were synthesized, one without iron in the reaction mixture and the other in the presence of 10 ppm ferric chloride. The solution was incubated at 37 °C with free air access and vigorous stirring for 5 days. The resulting black pigmented material was collected after centrifugation at 10,000 rpm for 10 min, then washed with 1% acetic acid and twice with deionised H_2O. Melanin was freeze-dried and stored in the dark at 4 °C until use. To prepare the sample for x-ray microscopy, synthetic NM was suspended in deionised H_2O at 4 mg/ml, then diluted 4-fold in 2% agarose gel. Once set, the gel was divided into cubes (approx. 8 mm^3) and subjected to the same ethanol dehydration, resin-embedding and sectioning procedure as described for PD tissue in part *A*. This enabled the thickness of synthetic NM deposits to be controlled, whilst also ensuring that they were subject to equivalent chemical exposure as intracellular NM in tissue.
It should be noted that this method attempted to synthesise only the (metal-binding) melanic portion of NM. Biological NM has a more complex structure, characterised by melanin, protein and lipid components, as well as metals such as iron (as discussed in depth in the Introduction) [2].

C. Reference materials

Dopamine and L-cysteine (precursor materials for melanin synthesis), along with alpha-synuclein and 2H-1,4-benzothiazin-3(H)-one (used as a model for the benzothiazine moiety present in NM) were purchased from Sigma Aldrich (Dorset, UK) as powders. Due to the water solubility of these materials, leaching of the sample material out of the matrix prevented agar gel embedding and ethanol dehydration, as performed for synthetic NM. 1 mg of each powder was instead spiked directly into resin and set overnight at 60 °C. 100- and 200 nm-thick sections were cut from the resulting block and air-dried onto copper TEM grids for STXM analysis, as described for the tissue preparation in Methodology part *A*. for the tissue sections. Type I horse spleen ferritin (Sigma Aldrich) was also used as an analogue for human ferritin. Ferritin was diluted in deionized H_2O to 1 mg/ml and deposited directly onto a silicon nitride membrane and allowed to dry for STXM analysis.

D. Masson-Fontana Staining for melanin

Tissue sections were stained for neuromelanin using Masson-Fontana silver nitrate stain. The staining protocol was iteratively adapted from that described by Bancroft and Gamble [3] for thin, resin embedded tissue sections. Ammoniacal silver nitrate solution (10% w/v, 3 μL) was deposited onto each section and left for 1 hour in the dark (a more rapid protocol than that used by Bancroft and Gamble, required for these ultra-thin tissue sections). Sections were then washed 3 times with distilled water and treated with 5% w/v sodium thiosulfate solution for 2 minutes before optical imaging. Reduction of silver nitrate to metallic silver by neuromelanin causes

a darkening of the pigment against background. A minimum-step protocol for positive silver nitrate staining of the thin, resin-embedded sections was developed. Counterstaining with nuclear fast red stain (as commonly performed as part of the Bancroft and Gamble staining protocol [3]) was eliminated to avoid associated damage to the integrity of the section, which was detrimental to subsequent optical imaging.

E. Optical Imaging

Optical imaging of the tissue sections before and after Masson-Fontana staining was performed using an inverted microscope (Olympus IX51) with a 40x objective lens. Images were acquired using a Q-Imaging QIClick™ Monochrome digital CCD camera with RGB filter for true colour and Q-Capture Pro 7 software.

F. X-ray spectromicroscopy measurements

STXM data were collected at the I08 beamline at Diamond Light Source (Oxfordshire, UK). Imaging was performed at spatial resolutions of 200 nm for whole cell maps and 100 nm for intracellular regions of interest, enabling chemical imaging analysis of sub-cellular components. Chemical speciation maps were created for features at the carbon *K*-edge (280-320 eV) and iron *L*-edge (700-740 eV) by acquiring paired images: one at an energy corresponding to a particular feature of interest, and one taken a few eV away from that. Subtracting the "off-peak" from the "on-peak" image produced a difference map, revealing the distribution of the feature of interest in isolation (for example Figures 2c, 2d and 2e). Background was subtracted using ImageJ Brightness and Contrast tool, where the background value was defined as the average pixel intensity from a blank resin region.

X-ray absorption spectra were collected by taking a series of images (termed a "stack") at x-ray energies across the carbon *K*-edge. Absorption spectra were generated for each pixel of every image such that distinct spectral features could be attributed to localised regions of interest. Transmitted x-ray absorption was normalised to incident beam I_0, then converted to optical density using a region of "blank" resin, i.e. devoid of sample material, thereby removing any background absorption artefacts. Similar to the speciation maps, the spectrum acquired from a blank resin region was then subtracted from a spectrum obtained from the sample material to display absorption features solely attributable to the sample [4].

Raw spectra acquired from powdered, water-soluble samples (alpha-synuclein, dopamine, L-cysteine and benzothiazine) were subjected to a 3-point boxcar average smoothing filter. This was principally for display purposes, since good signal-to-noise ratios were achieved up to 290 eV, the range where the primary features of interest for this study were observed. STXM data were processed using the aXis 2000 software package (http://unicorn.mcmaster.ca/aXis2000.html).

Estimates of the photon flux may be made based on values obtained during beamtime commissioning (typically ~10^9 ph/s at I08). However, radiation dose estimates need to take into account the requirement to determine accurately the x-ray absorption characteristics of both tissue and resin. An empirical approach was used here to confirm that spectral features are preserved at the carbon *K*-edge, the most sensitive to x-ray induced damage. The threshold at which photo-induced sample damage can occur, through over-exposure to the incident beam, was carefully determined in advance of each measurement by performing measurements of varying x-ray exposure on sample standards. Repeat stack measurements were performed on equivalent regions of neuromelanin to ensure that the level of beam exposure was below the threshold for inducing artificial features in the x-ray absorption spectra. In subsequent measurements, samples were protected by optimising the exit slit size for each measurement and minimising dwell time to <10 ms per point to ensure the dose was well below the damage threshold for all STXM data. Absorption spectra were acquired over a range of between 60 and 100 energy values, with a maximum energy resolution of 0.1 eV over absorption features of interest. Spatial resolution ranged from 100 nm to 350 nm, depending on the function and area of the region of interest. Preservation of absorption features, after repeat scans at 100 nm spatial resolution, is illustrated with an example for

WILEY-VCH

synthetic neuromelanin in Figure S7. Likewise, as previously described [4], no evidence of chemical reduction was observed in systematic repeated exposure of ferric iron standards prepared using the same resin embedding protocol.

G. Linear combination fitting and statistical analysis

Linear combination fitting of the intracellular NM spectrum after resin background removal was performed to ascertain how well the spectrum could be explained as a weighted sum of contributions from tissue-derived protein and synthetic NM. Fitting was performed with the Athena software in the Demeter package version 0.9 (http://bruceravel.github.io/demeter/), using a non-linear least squares fitting procedure. Spectra were subject to an edge step normalization prior to fitting and a χ^2 test was used to evaluate the quality of the fit.

Image cross correlation analysis was performed using the plugin, ImageCorrelationJ (http://www.gcsca.net/IJ/ImageCorrelationJ.html). As images were necessarily acquired at different spatial resolutions, correlation was performed after up-sampling of the lower resolution image in each pair, using the bilinear interpolation option in the ImageJ tool Resize.

H. X-ray fluorescence mapping

Nano-focus hard x-ray fluorescence maps were collected at the I14 beam line at Diamond Light Source (Oxfordshire, UK), using a 15 keV incident beam energy. Maps were acquired at 200 nm resolution over regions incorporating NM-rich neuronal cell bodies. Spectral fitting in PyMCA permitted plotting of intensity distributions for the individual elements present. An example of corresponding images for iron and sulfur distributions from a NM-rich neuron is shown in Figure S6.

Provider	ID	Neurological disorder	Age	Gender	STXM mapping of NM contrast	STXM spectral analysis of NM	Masson-Fontana silver nitrate stain of NM
CBTB	PD1	PD	73	Male	✓	✓	✓
NBTR	PD2	PD	79	Female	✓	✓	
NBTR	AD	AD	82	Male	✓	✓	
NBTR	Control	Control	85	Male	✓	✓	✓
CBTB	NBIA	NBIA	81	Female	✓		

Table S1: Cases used in the method development for this study, provided by NBTR and CBTB. PD: Parkinson’s disease; NBIA: clinicopathologically confirmed Neurodegeneration with Brain Iron Accumulation case of unknown genetic subtype; AD: Alzheimer’s disease; Control: no documented neurodegenerative disorder; STXM: scanning transmission x-ray microscopy.

WILEY-VCH

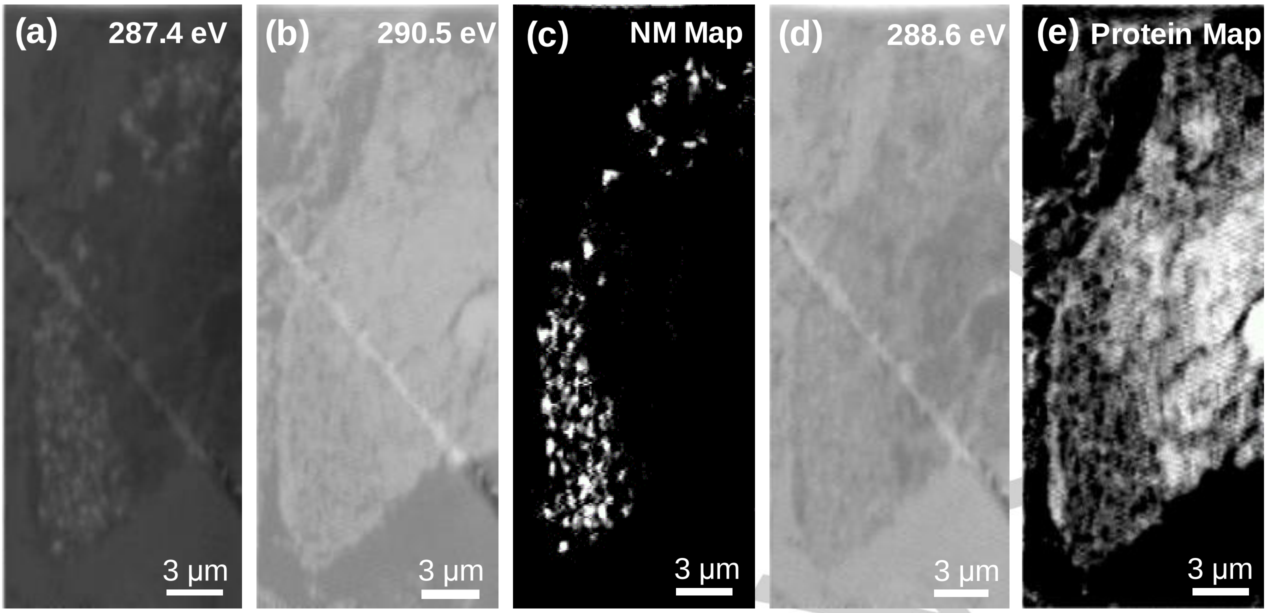

Figure S1: Creating STXM speciation maps for NM and protein distribution in a PD substantia nigra tissue section (200 nm thickness) from case PD1. **(a)** Image at NM peak (287.4 eV), **(b)** off-peak image at 290.5 eV, **(c)** speciation map showing distribution of intracellular NM, generated by subtracting image (b) from image (a). **(d)** Image at protein peak (288.6 eV), **(e)** speciation map showing distribution of tissue-derived protein, generated by subtracting image (b) from image (d). Brightness and contrast were manually adjusted in speciation maps for display purposes.

WILEY

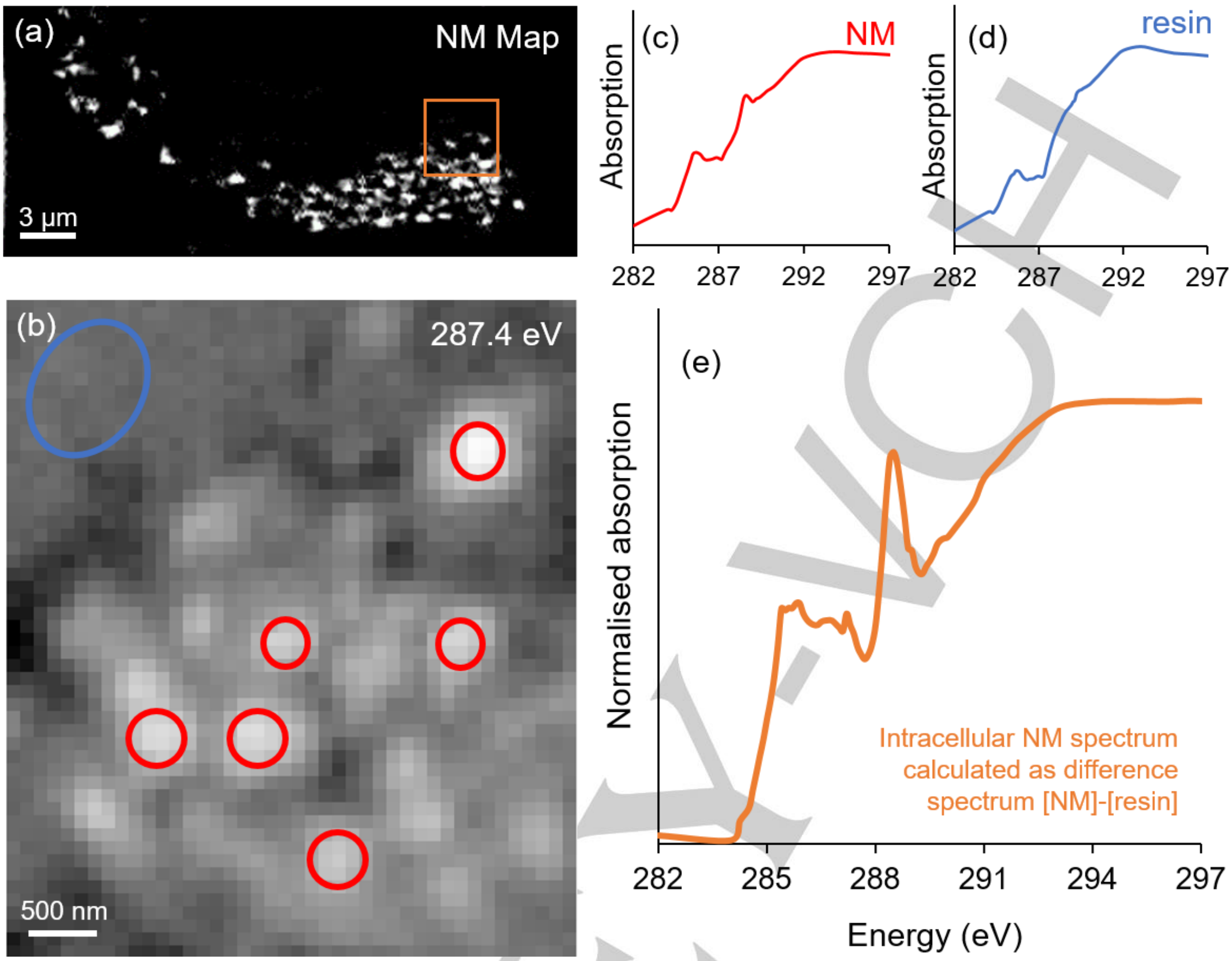

Figure S2: Obtaining site-specific absorption spectra for intracellular NM from a carbon *K*-edge stack. **(a)** NM speciation map of a dopaminergic neuron from case PD1, acquired at a spatial resolution of 200 nm, **(b)** image at NM peak (287.4 eV) from carbon *K*-edge stack. Images in the stack were acquired at 100 nm spatial resolution. Red markers show NM granules, blue marker shows blank resin region. Spectra were averaged from selected pixels for both NM and resin. **(c)** NM x-ray absorption spectrum, averaged over the six regions circled in red in (b), **(d)** resin x-ray absorption spectrum, averaged over the pixels circled in blue in (b), **(e)** absorption spectrum generated for intracellular NM by subtracting (d)[resin] from (c)[NM].

WILEY-VCH

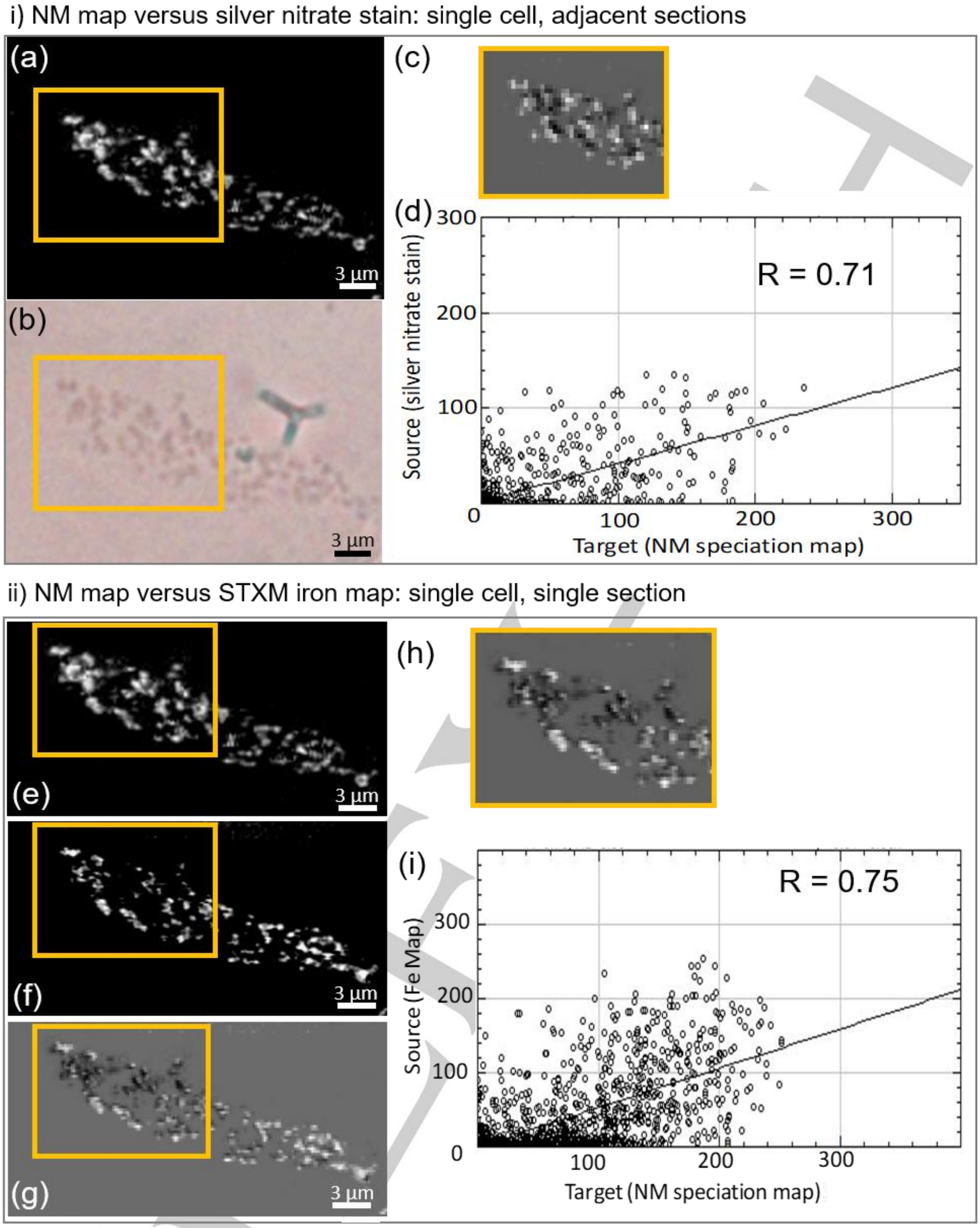

Figure S3: Image correlation for **i)** NM speciation mapping and silver nitrate staining in consecutive sections (200 nm thickness) of PD substantia nigra (case PD1), and for **ii)** synchrotron-acquired NM and iron maps of the same section. Average intensity from a local area size of 5 pixels was used to calculate the correlation coefficient. **(a)** NM speciation map (acquired at 200 nm resolution, adjusted using bilinear interpolation to 165 nm resolution), **(b)** silver nitrate stain (acquired at 165 nm resolution), **(c)** correlation map, where brightest spots show strongest correlation; silver nitrate stained image (b) was inverted using invert look-up table tool in Image J prior to correlation analysis. **(d)** Correlation plot for non-zero values in region defined in (a-b) where staining artefact was excluded, yielding a correlation coefficient of 0.71; **(e)** NM speciation map (acquired at 200nm resolution, adjusted using bilinear interpolation to 100 nm resolution); **(f)** iron map (acquired at 100 nm resolution); **(g)** correlation map for entire cell body; **(h)** correlation map for spatial region defined in (e-f); **(i)** correlation plot for non-zero values in region defined in (e-f), yielding a correlation coefficient of 0.75.

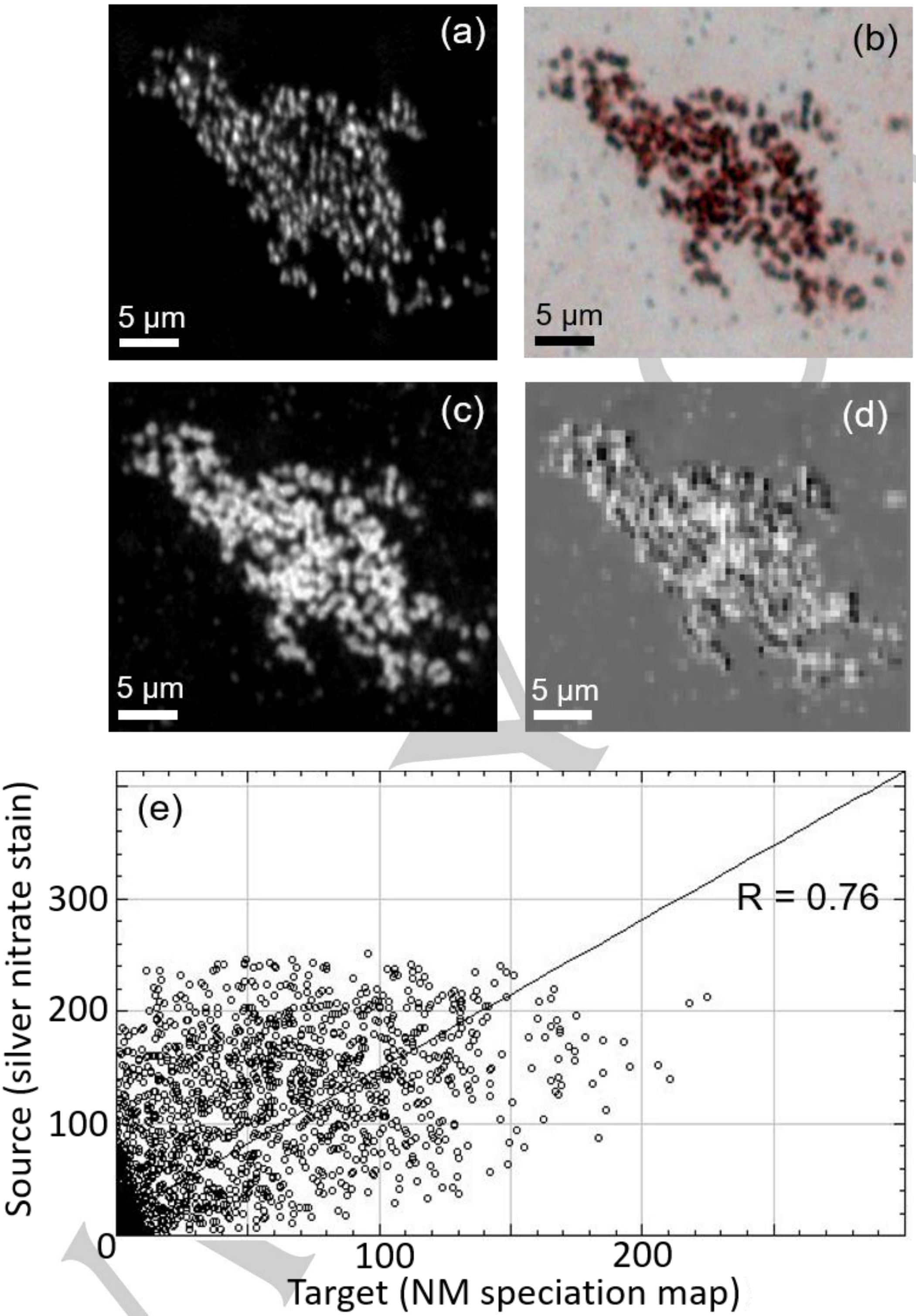

Figure S4: Image correlation for NM speciation mapping and silver nitrate staining in consecutive sections (200 nm thickness) of Control (neurologically healthy) substantia nigra. Average intensity from a local area size of 5 pixels was used to calculate the correlation coefficient. **(a)** NM speciation map (acquired at 200 nm resolution, adjusted using bilinear interpolation to 165 nm resolution), **(b)** silver nitrate stain (acquired at 165 nm resolution), **(c)** inverted stained image (b) using invert look-up table tool in ImageJ. Brightness and contrast tool used to threshold background. **(d)** Correlation map, where brightest spots show strongest correlation, **(e)** correlation plot for non-zero values, yielding a correlation coefficient of 0.76.

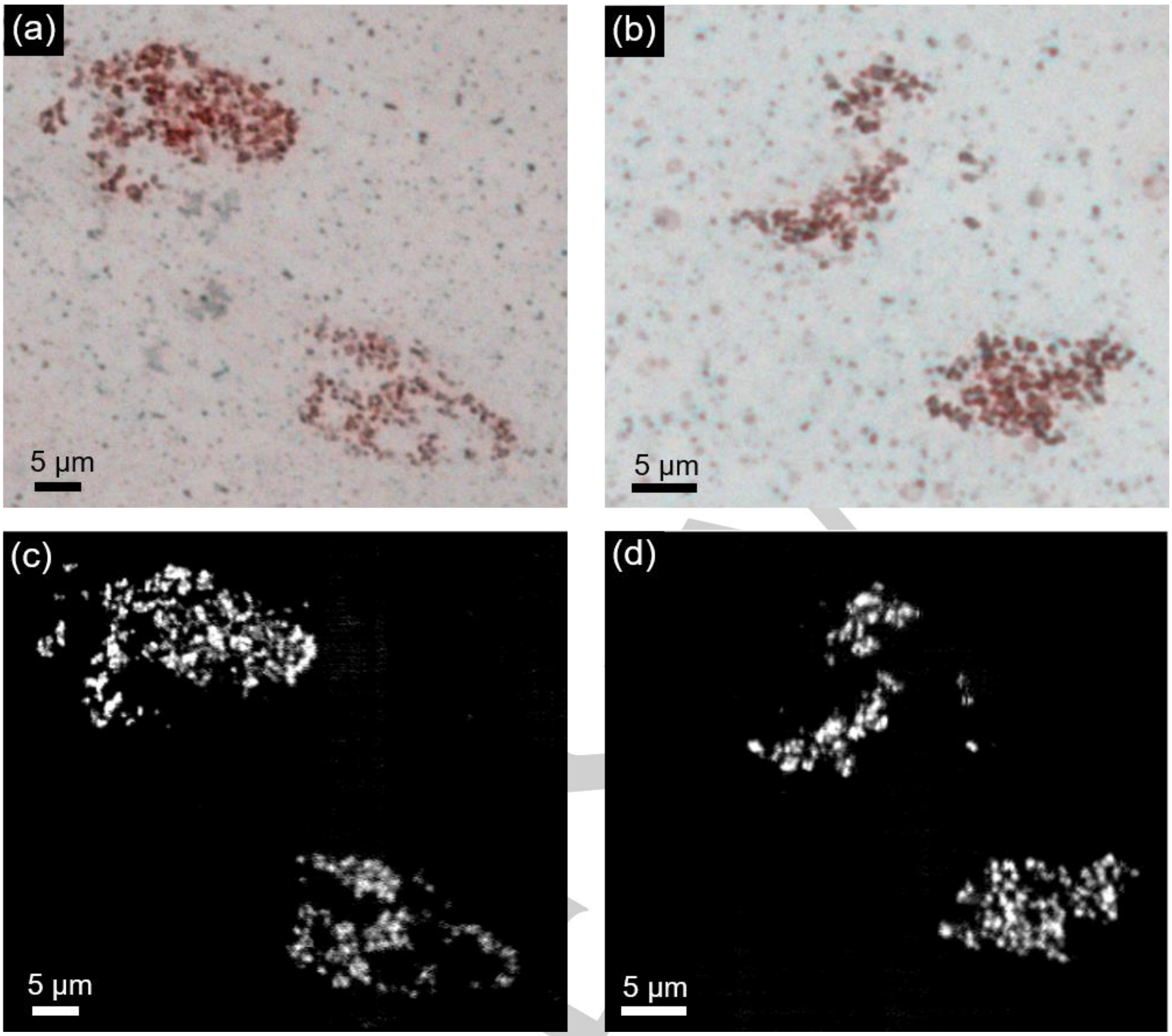

Figure S5: Additional example of NM silver nitrate staining paired with label-free NM mapping using STXM. **(a, b)** Silver nitrate-stained neurons in Control (neurologically healthy). **(c, d)** Paired neuromelanin STXM maps for a) and b), respectively. In this example, non-specific background staining in a) and b) precluded quantitative correlation analysis for this example.

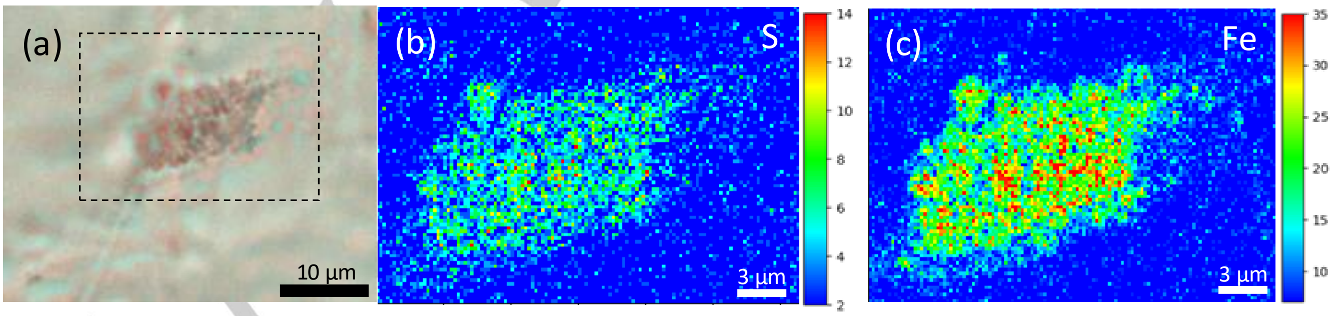

Figure S6: X-ray fluorescence maps of a NM-pigmented neuron in resin-embedded PD substantia nigra 3µm-thick tissue section. Maps were acquired at 200 nm resolution with a primary beam energy of 15 keV. **(a)** Light microscopy image, showing (inset, dashed line) the target NM-pigmented neuron in the PD tissue section; **(b)** sulfur distribution within the target neuron; **(c)** iron distribution within the target neuron.

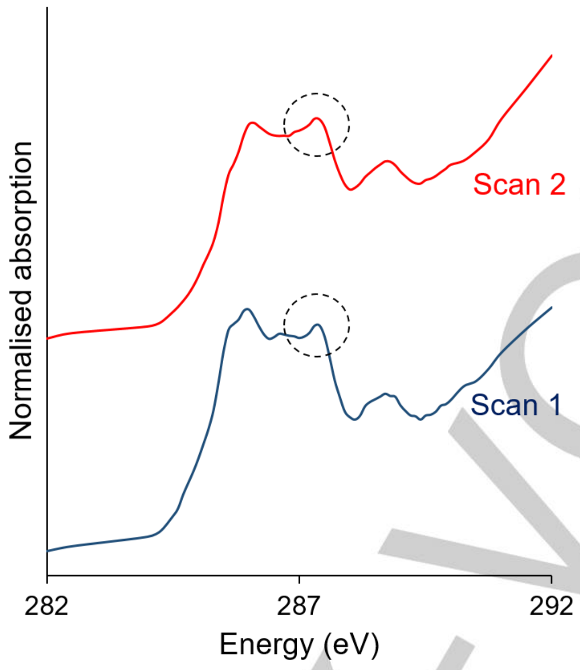

Figure S7: Repeat scans on synthetic NM (100 nm section thickness) at 100 nm spatial resolution, showing the extent to which the primary features are preserved; the feature used for NM mapping at 287.4 eV is circled.

References

[1] T. Shima, T. Sarna, H. M. Swartz, A. Stroppolo, R. Gerbasi, L. Zecca, *Free Radic. Biol. Med.* **1997**, *23*, 110-119.

[2] E. Ferrari, A. Capucciati, I. Prada, F. A. Zucca, G. D'Arrigo, D. Pontiroli, M. G. Bridelli, M. Sturini, L. Bubacco, E. Monzani, C. Verderio, L. Zecca, L. Casella, *ACS Chem. Neurosci.* **2017**, *8*, 501-512.

[3] J. D. Bancroft, M. Gamble, *Theory and practice of histological techniques*, 6th ed., Churchill Livingstone, London, **2008**.

[4] N. D. Telling, J. Everett, J. F. Collingwood, J. Dobson, G. van der Laan, J. J. Gallagher, J. Wang, A. P. Hitchcock, *Cell Chem. Biol.* **2017**, *24*, 1205-1215.

WILEY-VCH
